# Construction of an Omnidirectional Parametric Loudspeaker Consisting in a Spherical Distribution of Ultrasound Transducers

**DOI:** 10.3390/s18124317

**Published:** 2018-12-07

**Authors:** Marc Arnela, Oriol Guasch, Patricia Sánchez-Martín, Joan Camps, Rosa Ma Alsina-Pagès, Carme Martínez-Suquía

**Affiliations:** GTM Grup de recerca en Tecnologies Mèdia, La Salle, Universitat Ramon Llull, C/Quatre Camins 30, 08022 Barcelona, Catalonia, Spain; oriol.guasch@salle.url.edu (O.G.); patricia.sanchez@salle.url.edu (P.S.-M.); joan.camps@salle.url.edu (J.C.); rosamaria.alsina@salle.url.edu (R.M.A.-P.); carmenjulia.martinez@salle.url.edu (C.M.-S.)

**Keywords:** omnidirectional parametric loudspeaker, parametric acoustic array, nonlinear acoustics, omnidirectional sound source, ultrasound transducer

## Abstract

Omnidirectional sound sources are needed to perform a large variety of tests in acoustics. Typically, they consist of conventional speaker drivers arranged in a dodecahedron. However, the directivity of the speaker drivers sharpens with frequency, which induces an intense decrease of the sound pressure levels at the edges of the dodechaedron. In this work, the problem is mitigated by building an Omnidirectional Parametric Loudspeaker (OPL), which contains hundreds of small ultrasound transducers set on a sphere. Each transducer emits an ultrasonic carrier wave modulated by an audible signal. Thanks to nonlinear propagation, the air itself demodulates the signal bringing it back to the audible range. The construction of an OPL prototype is challenging. The structure has been built by 3D-printing a set of pieces that conform to the sphere. Each piece contains the exact location of the transducers, which are aligned in parallels to facilitate the structural assembly and the wiring. The performance of the OPL has been tested in an anechoic chamber. Measurements show that the OPL has a good omnidirectional behavior for most frequencies. It clearly improves the directivity of dodechaedral sources in the high frequency range, but performs worse at low frequencies.

## 1. Introduction

**Figure 1:**
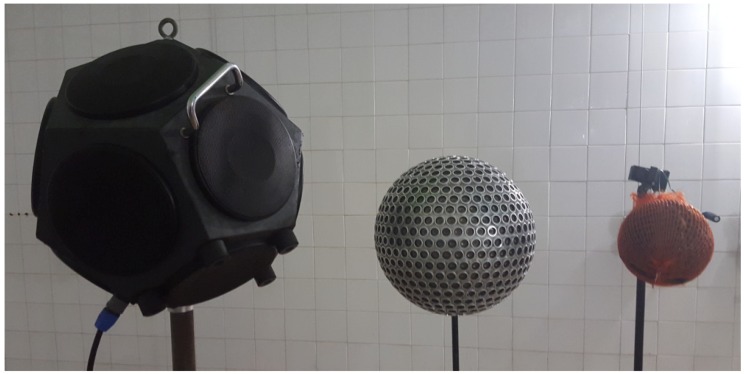
From left to right, commercial dodecahedral loudspeaker, new OPL and old OPL. The sound sources have, respectively, a diameter of 0.4 m, 0.25 m and 0.12 m.

Omnidirectional sound sources are needed for acoustic tests such as the measurement of the reverberation time in rooms [1], the measurement of the airborne sound insulation in buildings [2], or the measurement of the sound absorption coefficient in a reverberation room [3]. They are usually constructed by setting speaker drivers on the faces of a regular convex polyhedron so as to emit sound waves in all directions. While one may be tempted to think that the higher the number of faces in a regular polyhedron loudspeaker (RPL), the more omnidirectional the sound field will be, experimental tests have revealed that this is not always the case. For instance, in [4], it was shown that a tetrahedron (four faces) performed better than other higher order RPLs at the 4 kHz octave band. There is general agreement, however, that the dodecahedron RPL (12 faces, see the left source in Figure 1) is the one offering the best functioning when considering the whole audible frequency range up to 5 kHz. This was proven in [5], where an analytical model was developed to predict the acoustic radiation of RPLs.

It is well known that the directivity pattern of a conventional speaker driver sharpens with frequency [4,5,6]. This poses a cut-off frequency on the performance of the RPLs, beyond which the sources are no longer omnidirectional. Whereas one would expect to measure a constant sound pressure level (SPL) when following a circular path around the RPL, for most of them this is only true for frequencies lower than, say, the 500 Hz octave band. For higher frequencies, large SPL dips appear whenever the path crosses one of the vertices between faces. This poor functioning at the mid-high frequency range is common to all RPLs. This is why existing regulations for the characterization of omnidirectional sound sources tend to be rather permissive at high frequencies (see ISO 16283-1:2014 [2] and ISO 3382-1:2009 [7]). Nonetheless, this loss of omnidirectionality has been shown to be at the origin of some uncertainties in the measurement, for instance, of room impulse responses [8]. Moreover, one should bear in mind that, as quoted in [4], *the omnidirectional quality of a source should not be judged exclusively by its omnidirectional cut-off frequency or by the satisfaction of relaxed omnidirectional requirements at higher frequencies of interest*.

To try to remedy that situation, and get better omnidirectionality at high frequencies, several alternatives have been devised in literature. On the one hand, some authors have proposed to modify the measuring procedure with common dodecahedral sources, by rotating the source orientation in subsequent sets of measurements and changing the averaging process [9,10]. On the other hand, research has been conducted on constructing alternative source devices to RPLs. For instance, in the limit of very high frequencies, as those typically needed in scale model measurements, a sonic impulse can be generated through the expansion of a plasma ball, induced by heating a point in space with a pulsed laser beam [11]. Spark discharges [12] can also be used to that purpose and have been employed to measure the impulse responses in rooms [13]. Dielectric elastomer actuators [14] have been investigated as well to build up hemispheric [15] and semicylindrical sound sources [16]. An innovative design consisting of a powerful loudspeaker that feeds a small aperture through an inverse horn to concentrate and emit omnidirectional acoustic energy has also been designed for room acoustics [17].

The present work is to be also framed in the goal of exploring new devices to generate more omnidirectional sound fields from mid to high frequencies. The key idea is that of setting hundreds of small ultrasound piezoelectric transducers (PZTs) on a sphere, which emit audible sound thanks to the parametric acoustic array (PAA) phenomenon (see, e.g., [18,19]). In a nutshell, the PAA goes as follows. An ultrasonic wave (primary field) is emitted with a PZT, consisting of an ultrasonic carrier modulated with the desired audible signal. Thanks to nonlinear propagation effects in air, the primary field gets naturally demodulated, resulting in a strongly focused beam of audible sound (secondary field). As a consequence, most applications of the PAA to date have precisely been devoted to exploit the highly focused beams that can be generated through planar arrays of PZTs (see, e.g., [20] for a review). Recent developments, though, have extended the PAA to more complex geometries. In [21], for example, a flared horn was connected to an array of PZTs to enhance its directivity and intensity (see [22] for an analytical model of that device). A parametric beam focusing source was proposed in [23] which involved distributions of PZTs placed on a cylindrical shell (see also [24]). However, it was not until the work in [25] that it was first proposed to exploit the PAA to generate omnidirectional sound fields instead of focused ones.

This paper is to be viewed as a continuation of the ideas presented by some of the current authors in [25], where a first proof of concept of an omnidirectional parametric loudspeaker (OPL) was built (see the source in the right of Figure 1). As mentioned above, the central idea of the OPL consists in setting hundreds of PZTs on a sphere. Each PZT emits a strongly focused secondary (audible) beam. However, given that a high density of PZTs can be achieved on the spherical surface, we expect the acoustic field to exhibit a stronger continuity than that experienced when crossing a vertex in the circular path around a RPL.

The original OPL proof of concept in [25] suffered from severe limitations. Though promising, that was a handmade device consisting of a hollow polystyrene sphere with PZTs located at arbitrary positions, trying to fill the gaps. As such, the device was structurally weak and not useful for the practitioner. Moreover, it could not be replicated and a prediction model for it would prove useless because too many parameters were missing, starting with the precise location of the transducers on the sphere. The goal in this work has been to design and construct a new solid OPL prototype (see the central source in Figure 1), which overcomes many of the limitations of the previous one. The dimensions of the OPL and PZT distribution have been analyzed by means of the theoretical model in [26], in order to find an appropriate balance between the acoustic performance and the circuitry and construction requirements. In addition, a 3D printed casing has been designed to have a precise control of the PZT locations and to get structural stiffness. The spherical casing consists of several pieces, which facilitates the OPL assembly and the electrical connections. It includes a hole for a microphone stand as well. The directivity of the new constructed OPL prototype has been measured in an anechoic chamber. The results are compared to those obtained with a commercial dodecahedral loudspeaker and discussed in terms of existing regulations. A preliminary version of some of the contents in this work was presented in [27].

The paper is structured as follows. Section 2 provides an overview of the PAA theory, which is at the basis of the OPL functioning. Section 3 describes the new OPL prototype, including the design, the construction process and the associated circuitry. Section 4 shows the experimental results carried out in an anechoic chamber, to test the behaviour of the new OPL. The conclusions are provided in Section 5.

## 2. Foundations of the Parametric Acoustic Array

The functioning of the OPL relies on exploiting nonlinear wave propagation in air through the PAA phenomenon. This was first proposed in Westervelt’s seminal paper [18]. Westervelt derived the following wave equation to describe sound propagation in a thermoviscous fluid,

(1) $$\begin{array}{c} \frac{1}{c_{0}^{2}}\frac{\partial^{2}p}{\partial t^{2}}-\nabla^{2}p=\frac{\delta }{c_{0}^{4}}\frac{\partial^{3}p}{\partial t^{3}}+\frac{\beta }{\rho_{0}c_{0}^{4}}\frac{\partial^{2}p^{2}}{\partial t^{2}}, \end{array}$$

where *p* stands for the acoustic pressure. One can recognize the left-hand side (l.h.s) of Equation (1) as the standard lossless linear wave equation, $c_{0}$ designating the speed of sound. In turn, the first term in the right-hand side (r.h.s) of Equation (1) accounts for the air thermoviscosity, δ being the sound diffusivity parameter. The second term in the r.h.s is the nonlinear one. $\rho_{0}$ denotes the ambient density and β the nonlinearity parameter.

In the case of weak nonlinearity, one may attempt a quasilinear approximation to Equation (1) (see, e.g., [18,19]). The acoustic pressure *p* can be decomposed into a primary field $p_{1}$ that satisfies the linearized version of Equation (1), plus a secondary field, $p_{2}$, which arises as a nonlinear byproduct of $p_{1}$. Inserting $p=p_{1}+p_{2}$ ($\left|p_{2}\right|\ll \left|p_{1}\right|$) into Equation (1) provides two linear equations respectively governing the primary and secondary acoustic fields,

(2a) $$\begin{array}{c} \frac{1}{c_{0}^{2}}\frac{\partial^{2}p_{1}}{\partial t^{2}}-\nabla^{2}p_{1}-\frac{\delta }{c_{0}^{4}}\frac{\partial^{3}p_{1}}{\partial t^{3}}=0, \end{array}$$

(2b) $$\begin{array}{c} \frac{1}{c_{0}^{2}}\frac{\partial^{2}p_{2}}{\partial t^{2}}-\nabla^{2}p_{2}-\frac{\delta }{c_{0}^{4}}\frac{\partial^{3}p_{2}}{\partial t^{3}}=\frac{\beta }{\rho_{0}c_{0}^{4}}\frac{\partial^{2}p_{1}^{2}}{\partial t^{2}}. \end{array}$$

It can be easily checked that the Fourier transform of Equation (2a) yields a Helmholtz equation, $-\nabla^{2}\hat{p}-k^{2}\hat{p}=0$, with complex wavenumber $k\approx k_{0}\left[1-i(\delta \omega /2c_{0}^{2})\right]$, and with $\hat{p}$ standing for the Fourier transform of *p*, ω for the angular frequency, $k_{0}=\omega /c_{0}$ for the real wavenumber and $i=\sqrt{-1}$ (see, e.g., [26,28]). The solution to that equation simply consists of propagating plane waves with attenuation factor $\alpha =\delta \omega^{2}/2c_{0}^{3}$. Once squared, these waves will act as a source term for the secondary field equation (2b).

The essentials of the PAA can be understood as follows. Consider a PZT emitting two collimated plane waves in the positive *z*-axis (primary field),

(3) $$\begin{array}{c} p_{1}=P_{1}e^{-\alpha_{1}z}\sin\left(\omega_{1}t-k_{1}z\right)+P_{2}e^{-\alpha_{2}z}\sin\left(\omega_{2}t-k_{2}z\right), \end{array}$$

with $\alpha_{i}=\delta \omega_{i}^{2}/2c_{0}^{3}$, i=1,2 representing each wave attenuation factor. Squaring Equation (3) to get $p_{1}^{2}$, and inserting it into Equation (2b), results in a first term that involves twice the emitted frequencies $2\omega_{1}$ and $2\omega_{2}$, a second term with the frequency summation $\omega_{1}+\omega_{2}$ and a third one including the difference frequency $\omega_{d}=\left|\omega_{1}-\omega_{2}\right|$. If one selects $\omega_{1}$ and $\omega_{2}$ to be close together and to lie in the ultrasonic range, the difference frequency component of the secondary field $p_{2}$ will be audible. For instance, choosing $\omega_{1}=40$ kHz and $\omega_{2}=41$ kHz would result in an audible difference frequency component of 1 kHz. This is the key point behind PAA applications, where the primary field typically consists of an ultrasonic carrier wave modulated with a broadband signal in the audible range (see, e.g., [29,30]).

The difference frequency component is the one that matters for the PAA (the other components will lie in the ultrasonic range and get rapidly absorbed). If we insert Equation (3) in the source term of Equation (2b) and solve the equation using the standard Green function approach, we recover Westervelt’s celebrated expression for the audible secondary pressure, $p_{d}$, at a far-field point $\left(r_{0},\theta_{0}\right)$ (see, e.g., [18,19,31]),

(4) $$\begin{array}{c} p_{d}\left(r_{0},\theta_{0}\right)=KS_{0}P_{1}P_{2}D_{W}\left(\theta_{0}\right)\cos\left(\omega_{d}t-k_{d}r_{0}-\phi \right), \end{array}$$

with

(5) $$\begin{array}{c} K=\frac{\beta \omega_{d}^{2}}{4\pi \rho_{0}c_{0}^{4}r_{0}\left(\alpha_{1}+\alpha_{2}\right)},\;\;D_{W}\left(\theta_{0}\right)=\frac{\alpha_{1}+\alpha_{2}}{\sqrt{{\left(\alpha_{1}+\alpha_{2}\right)}^{2}+k_{d}^{2}{\left(1-\cos\theta_{0}\right)}^{2}}},\;\;\tan\phi =\frac{k_{d}\left(1-\cos\theta_{0}\right)}{\alpha_{1}+\alpha_{2}}. \end{array}$$

In the above expressions, $r_{0}$ represents the distance from the PZT to the observer and $\theta_{0}$ the sustained angle between the PZT emission axis and the observer’s location. $k_{d}=\omega_{d}/c_{0}$ is the wave number of the difference frequency wave. The factor $D_{W}\left(\theta \right)$ is known as the Westervelt directivity and is responsible for a sharp secondary audible field without lobes, which is at the core of the audio spotlight applications [30]. As observed from *K* in Equation (5), the PAA effect strongly increases with frequency, an associated problem thus being the generation of intense pressure fields at low frequencies. Given that $p_{d}$ is a nonlinear by-product of the primary field, the latter needs to be very powerful to get admissible audible sound levels for practical applications.

Equations (4) and (5) reflect the basics of the PAA phenomenon. However, they suffer from some limitations when applied to determine the far field pressure of parametric loudspeaker arrays (PLAs). The main one is that of assuming collimated primary waves. Through the past decades, several models have been proposed to better match the theoretical predictions with measurement results. The first step was to incorporate the product of the primary field directivities into Equation (4) (see, e.g., [31] and also [20,32] for a recent review on further approaches). Quite recently, a convolution model was developed to deal with PLAs whose PZTs could have beam widths up to $80^{\circ }$ [33]. The model suggests the following prediction formula for the acoustic far field, (to be compared with Equation (4)),

(6) $$\begin{array}{c} p_{d}\left(r_{0},\theta_{0}\right)=K\cos\left(\omega_{d}t-k_{d}r_{0}\right)\int_{-\psi_{0}}^{\psi_{0}}D_{1}\left(\psi \right)D_{2}\left(\psi \right)D_{W}\left(\theta_{0}-\psi \right)\;d\psi . \end{array}$$

In Equation (6), $\psi_{0}$ denotes half of the PZT beam width. As seen from the equation, the directivity of the audible difference frequency field is computed by making the convolution of Westervelt’s directivity and the product of the primary field directivities, namely $D_{1}\left(\psi \right)$ and $D_{2}\left(\psi \right)$. It was reported in [33] that, for planar PLAs, the convolution model better agrees with experimental results than previous models. A short while ago, the model has been extended to deal with PLAs set on curved surfaces [26], which could prove useful to analytically model the performance of several non-conventional PLA devices [21,23,24]. In particular, the generalized convolution model in [26] has been used as a guidance for designing the OPL prototype presented in this work.

## 3. Construction of an Omnidirectional Parametric Loudspeaker

### 3.1. Design

As explained in the Introduction, an OPL consists of a sphere with hundreds of ultrasound PZTs on it. The design of such a device depends on several choices. One has to select a PZT model, determine the radius of the sphere and distribute the transducers on its surface. The goal is to obtain an omnidirectional sound source in the widest possible frequency range. Moreover, the OPL should be moderate in size to minimize costs (the larger the prototype the larger the number of transducers), as well as light and handy enough to perform in situ acoustic tests.

To start with, we shall select an ultrasound PZT among all available options in the market. After testing many of them, we decided to use the Multicomp 16 mm Transmiter (ST160) [34], which has a base radius of $r=8\times 10^{-3}$ m and produces high sound pressure levels in the audible range. Moreover, it has an economic price.

The next step is finding a suitable distribution of the ST160 transducers on the sphere to achieve the required omnidirectionality. Distributing equally spaced points on the surface of a sphere is a notable, not simple, classical problem (see, e.g., [35]). In [26], two options were considered to address it. Let us briefly summarize them here.

**Figure 2:**
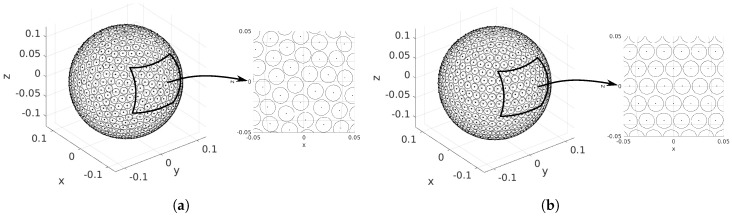
Distribution of the ST160 ultrasound transducers on the OPL for (**a**) the force equilibrium solution and (**b**) the equal-area partitioning approach. The radius of the sphere is set to R=0.125 m. The transducer base is represented with circles of radius $r=8\times 10^{-3}$ m.

The first option either consists of minimizing an associated energy problem (see, e.g., [35,36]) or in finding the solution to its force equilibrium counterpart (see [37]). Figure 2a shows the optimal solution for the latter when considering a sphere radius of R=0.125 m (see below for that choice) and the ST160 transducers. A total number of 755 PZTs fit on the sphere surface, with a distance of $\Delta r=0.1\times 10^{-3}$ m between them to prevent contact. The figure reveals the main inconvenience of such an optimal distribution: the practical construction and inner wiring of the PZTs would be very complex because of the lack of symmetries in the PZT locations.

As for the second option, known as the equal-area partitioning method [26,35], the sphere is divided into strips having the width of the ST160 diameter. The strips are spherical parallel segments, and their number equals the maximal number of PZTs that fit in a sphere meridian. Each strip is filled with PZTs, with their center on the mid parallel, and keeping the same clearance between PZTs as for the force equilibrium case. Figure 2b shows the equal-area partitioning distribution of the ST160 transducers in the same sphere of radius R=0.125 m. Note that this strategy aligns the transducers in parallels, which greatly simplifies the circuitry and welding process, and also helps the design and construction of the spherical casing where the PZTs are placed. However, this is at the expense of reducing the total number of transducers to 750, as opposed to the 755 of the force equilibrium solution.

Nonetheless, the two approaches were contrasted in [26] using the convolution model on curved surfaces outlined in Section 2. The simulations showed a very omnidirectional secondary field for both distributions. Consequently, the equal-area approach was selected for the OPL prototype given its advantages for construction.

Once having a PZT model and a distribution type, it becomes necessary to determine a proper radius for the sphere. At this point, one should bear in mind that the acoustic pressure measured at a fixed distance from a hemisphere containing, say *N* transducers, will undoubtedly be less than that emitted from a planar array with the same number of transducers. Not only that, but even for an infinite planar array, there exists an asymptotic upper limit to the sound pressure level one could measure at a fixed distance from it. This is because of the high directivity of the sound emitted by each PZT. The sound of distant transducers will never reach a point that is out from their emission cone. The situation is obviously more drastic for the transducers set on a sphere because of curvature. This is clearly exemplified in Figure 13 of [26]. In that figure, one can observe how, beyond a certain point, slightly increasing the radius of an OPL results in a huge augmentation of the number of PZTs, but only in very little gain of acoustic pressure. For instance, modifying the radius of the OPLs in Figure 2 from R=0.125 m to R=0.15 m would approximately increase the number of PZTs from 750 to 1500, but barely improve the generated acoustic field in 2 dB. Therefore, selecting a radius for the OPL involves a trade-off between the acoustic efficiency, the number of transducers (and consequently the final price), the construction facilities (e.g., casing and wiring) and the practical handling (weight and size).

To summarize, and in view of all the above considerations, our final choice for the prototype has consisted of a sphere of radius R=0.125 m with 750 PZTs (ST160) distributed according to the equal-area partitioning approach.

### 3.2. Spherical Casing

**Figure 3:**
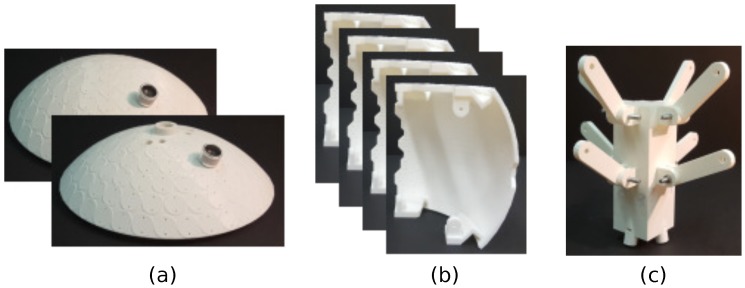
3D-printed pieces to build the OPL. (**a**) spherical caps; (**b**) lateral sectors and (**c**) central piece.

According to the design in the previous section, 750 transducers have to be set on a spherical casing. A first requirement for its construction is thus robustness. The OPL prototype has to be stiff enough to resist the weight of the transducers and user manipulation, without being deformed. Moreover, its interior has to be accessible for wiring and soldering all the PZTs. To accomplish these goals, we have resorted to 3D-printing six rigid plastic pieces that constitute the OPL surface, plus an inner component that provides structural stability and helps with assembling all components (see Figure 3).

**Figure 4:**
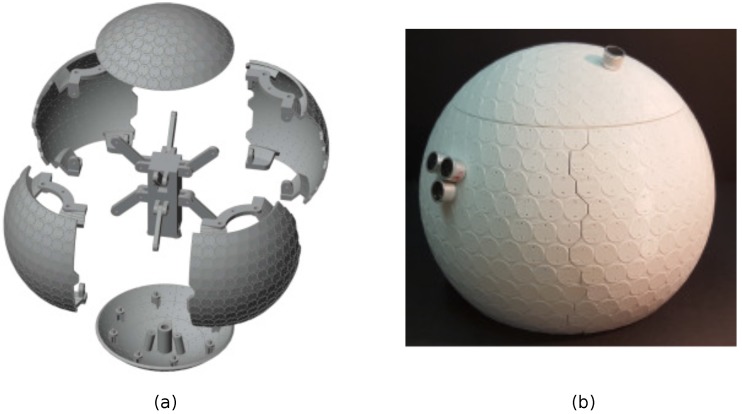
(**a**) assembly scheme of the printed OPL components; (**b**) assembled OPL with some sample PZTs.

The pieces that form the OPL outer sphere are divided into two spherical caps and four lateral sectors. Thanks to the PZTs parallel arrangement in the equal-area partitioning scheme, the spherical caps can be easily delimited (see Figure 3a). On the contrary, the lateral sectors present more problems because the PZTs are not aligned along a meridian. The sectors have therefore been delimited with polygonal edges to prevent them from sharing a transducer (see Figure 3b). On the other hand, the inner central piece works as the backbone of the structure (see Figure 3c). It connects the six outer pieces through bars and screws, and prevents them from collapsing. Moreover, it allows us to support the OPL with a microphone stand, which enters the structure through a hole on the bottom spherical cap that is connected to this central piece, ensuring a high stability. Figure 4a displays the assembly strategy of all components and Figure 4b shows the assembled pieces with only a few sample PZTs on it.

Another requirement is that the PZTs have to be accurately positioned on the sphere, according to the design, to ensure omnidirectionality and to simplify the wiring. To that purpose, circular plateaus have also been printed on the six outer pieces, regarding where to place the PZTs. Two holes are left on each one to insert the pins of the transducers (see Figure 4b).

Finally, let us mention that the heat dissipation of the OPL is minimal, making any cooling system unnecessary.

### 3.3. Circuitry

**Figure 5:**
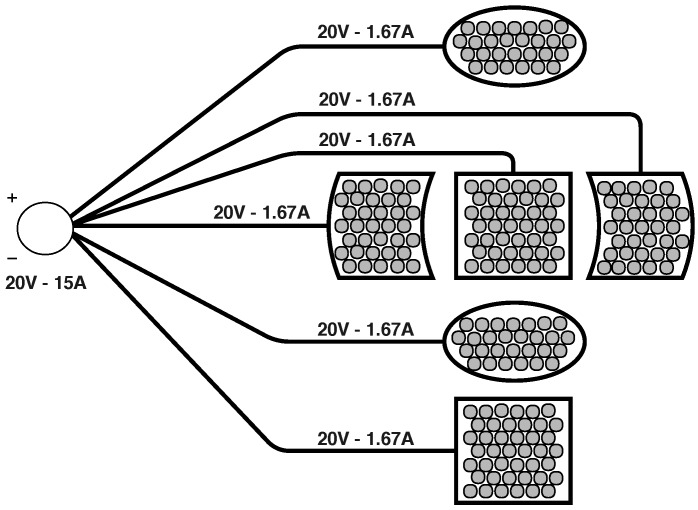
Scheme of the internal wiring of the OPL.

As detailed in the previous section, the spherical casing of the OPL consists of six pieces. These have been star-form electrically connected with a central concentrator in the OPL, which in turn becomes connected to the external power supply, as shown in Figure 5.

All the PZTs of the OPL have been connected in parallel resulting in a real equivalent impedance of the entire loudspeaker of approximately 2Ω. This impedance value is suitable for the working margin of the amplifier, which ranges from 2 to 8 Ω. The maximum power dissipated by the OPL is therefore $\frac{V_{eff}^{2}}{Z}=200$ W, where $V_{eff}$ stands for the rms voltage and *Z* corresponds to the complex-valued generalization of the resistance. The maximum current needed for the entire set is 10 A.

The current that flows through each piece is about one sixth of the total current (i.e., ∼1.67 A), as described in Figure 5. It is to be noted, however, that all six pieces are not identical; the four lateral ones contain almost the same number of transducers, but the two spherical caps host a few more ones.

**Figure 6:**
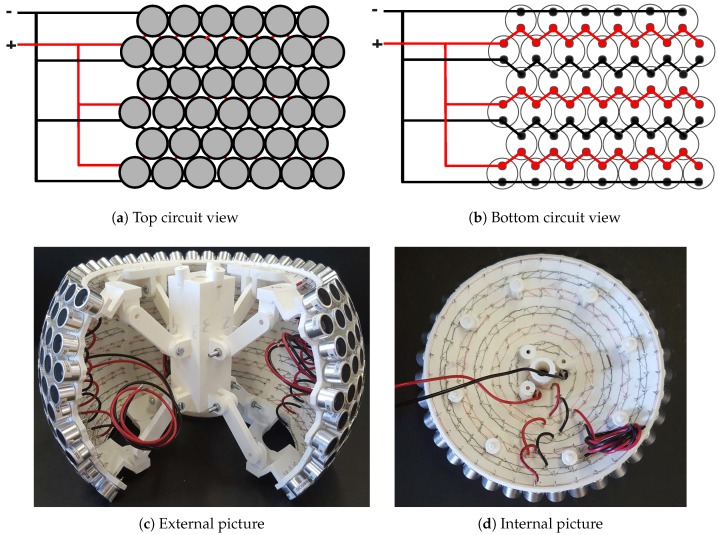
Schemes and pictures showing the zigzagged naked wiring carried out in each piece to connect all the transducers.

Each piece of the OPL contains more than 100 PZTs aligned in parallel rows, as described before, which strongly simplifies the circuitry. This is schematically depicted in Figure 6a,b, which respectively show a top and bottom view of the circuitry design. A picture of three assembled pieces including the PZTs and the wiring is presented in Figure 6c, while that of one of the spherical caps is pictured in Figure 6d. A naked cable $0.2\;{\mathrm{mm}}^{2}$ in the section was selected for the inner connection of the PZTs, which admits up to 15 A (see [38] for details). This surpasses the need of ∼1.67 A per piece, yet circumvents any potential problem due to the different number of PZTs in the pieces [39]. The naked wire was soldered following a zigzag pattern to minimize the length of the wire and to facilitate the welding process. To supply the power to the central concentrator that gathers the wiring of the assembly, a $1\;{\mathrm{mm}}^{2}$ section cable was chosen that accepts up to 20 A [39] (see also [40] for further details).

## 4. Experimental Testing in an Anechoic Chamber

### 4.1. Experimental Setup

**Figure 7:**
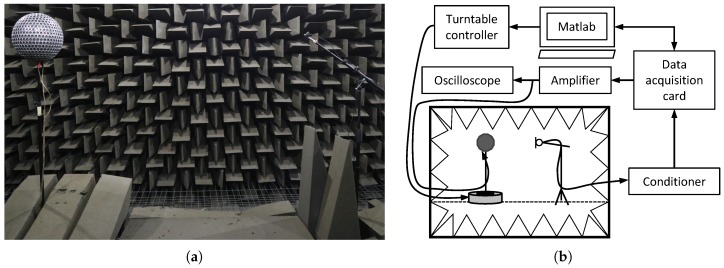
Experimental setup. (**a**) picture of the OPL measuring set in the anechoic chamber; (**b**) sketch of the data acquisition setup.

The directivity of the OPL was measured in the anechoic chamber of La Salle, Universitat Ramon Llull (see Figure 7a). The chamber has a free volume of 215 $m^{3}$ and an anechoic cut-off frequency of about 70 Hz. For comparison purposes, a commercial dodecahedral loudspeaker (CESVA BP012) was also characterized under the same conditions. Figure 7b contains a diagram of the experimental setup used to measure the directivities of the OPL and the dodecahedral loudspeaker. The sound source under test was located on top of a computer-controlled turntable using a narrow stand. Thick acoustic foam was used to cover the upper surface of the turntable to reduce sound reflections and scattering effects (see Figure 7a). We positioned a 1/4 inch free-field microphone (G.R.A.S. 40BF) at a fixed distance of 1.5 m from the sound source center, as required by the ISO 3382-1 [7] to characterize omnidirectional sound sources. The protection cover of the microphone was removed to extend its flat frequency response at higher frequencies. The microphone was connected to a conditioning amplifier (B&K Nexus) and its output to a Data Translation 9832 card. The same card was employed to generate the excitation signal. This signal became amplified using an Ecler XPA3000 with an input voltage of 17 Vpeak and next driven to the sound source. This voltage value was selected to avoid damaging the ultrasound transducers of the OPL (<20 Vpeak). This value was configured by tuning the volume of the amplifier while measuring with an oscilloscope the voltage driven to the sound source. The experimental setup was controlled using a custom software developed in Matlab (R2010b, The MathWorks, Inc., Natick, MA, USA).

The turntable was configured to continuously rotate in the azimuth angle. A sinusoidal signal was generated to measure the directivity at single frequencies. These frequencies were chosen as the central frequencies of the 1/1 octave bands from 125 Hz to 8 kHz. The dodechaedral loudpspeaker directly emitted these signals. However, for the OPL, each sinusoidal signal was modulated with an Upper Side Band Amplitude Modulation (USBAM). The carrier was selected to have a frequency of 41 kHz. Remember that, thanks to nonlinearity in air (see Section 2), the emitted signal is demodulated, bringing the sinusoidal signal back to the audible range.

### 4.2. Results

**Figure 8:**
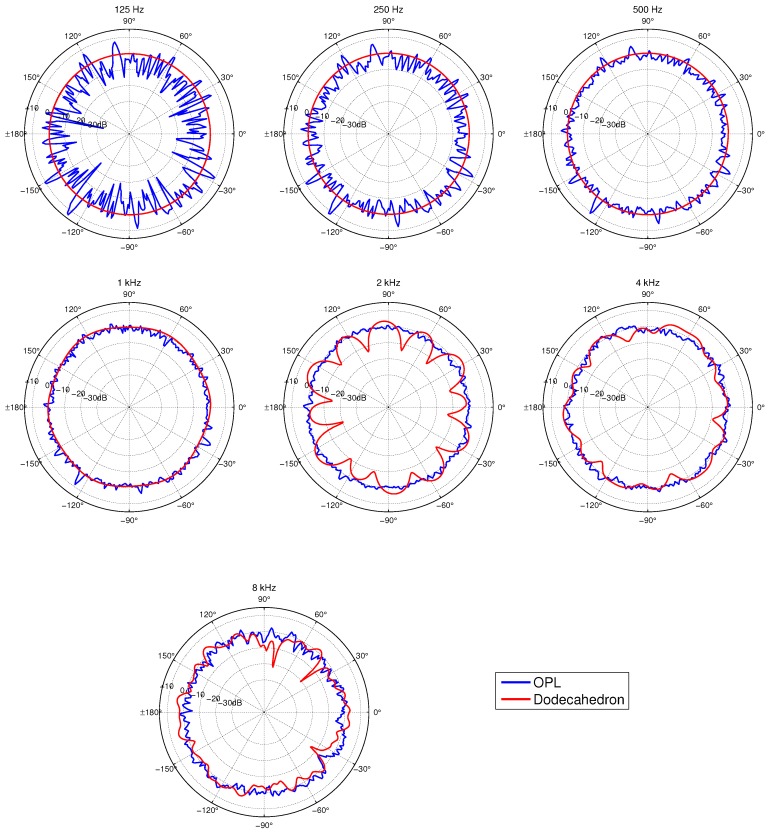
Directivity pattern of the OPL (blue line) and a dodecahedral loudspeaker (red line) obtained when emitting sinusoidal signals at different frequencies. Results are normalized with respect to the average sound pressure level.

Figure 8 presents the directivity patterns measured for the OPL and the dodecahedral loudspeaker. The results are represented in a polar graph with a $1^{\circ }$ of resolution, and normalized with the mean sound pressure level at each frequency.

As can be observed, the OPL has a similar directivity pattern for frequencies above 500 Hz, presenting only some small oscillations that tend to diminish with increasing frequency. However, parametric acoustic arrays have difficulties to reach high SPL values for low frequencies (below 500 Hz) (see Section 2). This reflects in the large oscillations of the OPL directivity pattern. On the contrary, the dodecahedral loudspeaker presents a radically different behaviour. Below 500 Hz, the dodecahedral loudspeaker is very omnidirectional, since almost identical sound pressure levels are measured when rotating the sound source. However, as the frequency increases (above 1 kHz), some strong dips manifest in the sound pressure levels at given angles. The dips appear because the directivity of each speaker driver sharpens with frequency, generating acoustic shadows in the vicinity of the edges between the dodecahedron faces. Note also that the onset of these dips clearly depends on frequency, the directivity pattern at 2 kHz being the most critical one. In contrast, and as observed before, the OPL presents a more uniform directivity in this frequency range thanks to the high density of transducers on its surface, though it performs clearly worse for lower frequencies.

It is also interesting to analyze the Directivity Index (DI) defined in the ISO 3382-1 [7], needed to qualify the omnidirectionality of a sound source. This can be computed as

(7) $$DI_{i}=L_{360^{\circ }}-L_{30^{\circ },i},$$

with $L_{360^{\circ }}$ and $L_{30^{\circ },i}$ standing for the mean pressure level in the range $\phi ={\left[0,360\right]}^{\circ }$ and along a $30^{\circ }$ arc, respectively. The index i=[0,360] is used to denote each one of the $30^{\circ }$ arcs. Note that the DI is nothing else than a $30^{\circ }$ average of the directivity pattern along the azimuth angle. However, the ISO 3382-1 requires one more averaging—this time in frequency. Octave band levels should be measured when the sound source is excited with a pink signal. This would result in a larger smoothing of the strong level decays observed in Figure 8 for the dodechaedral loudspeaker. We have then estimated the DI assuming the single frequency directivity patterns of Figure 8. We believe that, in this way, a fairer comparison between the sound sources may be obtained, since only one averaging (in azimuth) is applied. On the other hand, we acknowledge that 1/1 octave band levels could be obtained by using a pink noise to excite the two sound sources. However, this signal is inefficient for the OPL because it distributes the energy among several frequencies, resulting in a large reduction of the OPL emitted sound pressure levels. This could be circumvented with input signals based on frequency swept signals [41]. Nonetheless, the OPL performance with such excitations, or others like maximum length sequence signals (MLS), is beyond the scope of this paper, and will be examined in future works.

**Figure 9:**
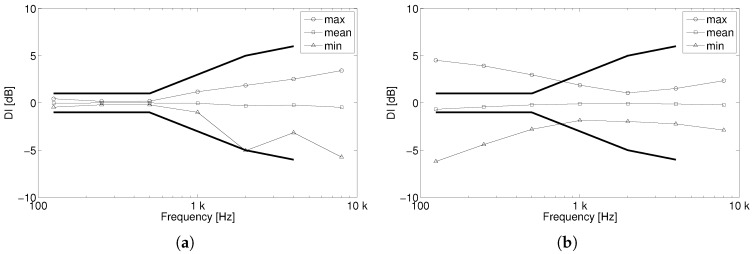
Directivity Index (DI) deviations for (**a**) the dodecahedral loudspeaker and (**b**) the OPL, based on the ISO 3382 when sinusoidal signals are emitted. The ISO 3382 limiting values are represented with thick lines.

The DI obtained for the OPL and the dodecahedral loudspeaker is shown in Figure 9. The maximum and minimum values for each frequency are represented in the figure, as usually done by omnidirectional sound source manufacturers to show compliance with the ISO requirements. Moreover, the arithmetic mean of the DI is plotted for completeness. Thick lines are used for the maximum and minimum DI limiting values specified by the ISO 3382-1.

If we first focus on the dodecahedral loudspeaker (see Figure 9a), we can observe that the DI deviations increase with frequency. Indeed, this index follows the directivity patterns shown in Figure 8, which are more omnidirectional for the lower frequencies than for the higher ones, resulting in smaller DI values for the former. Note also that, at 2 kHz, the minimum DI has a larger deviation than their neighbour frequencies, since the directivity pattern at 2 kHz (see Figure 8) is the one presenting the largest dips. As expected, this sound source fulfills with the ISO 3382-1 requirements. It is also to be mentioned that smaller DI deviations would be produced with a pink noise, since, as discussed before, this would smooth the strong decays produced in the directivity patterns at single frequencies.

Regarding the OPL (see Figure 9b), its DI presents the opposite behaviour than that of the dodecahedral loudspeaker. It has smaller values for the higher frequencies than for the lower ones. Above 1 kHz, the DI shows little variation with frequency, as also observed in the directivity patterns of Figure 8. The OPL does not fulfill the requirements of the ISO 3382-1 in the low frequency range. Nevertheless, one should bear in mind that the ISO requirements are better suited to the behaviour of the dodecahedral loudspeaker than that of the the OPL. They are much more restrictive at low frequencies, where dodecahedral loudspeakers are very omnidirectional, but much more permissive in the high frequency range, where these sound sources clearly fail and the OPL performs better (see also Figure 8).

For completness, Table 1 contains the second quartile (Q2), the deviations from Q2 of the first and third quartiles (Q1 and Q3), and the minimum and maximum values of the directivity patterns shown in Figure 8 without normalization, for both, the dodecahedral loudspeaker and the OPL. Moreover, this table also provides the energetic mean sound pressure level $L_{\mathrm{mean}}$ used to normalize the directivity patterns. At this point, it is worth mentioning that neither the OPL nor the dodecahedral loudspeaker worked at their very highest power level, in order to avoid damaging the OPL prototype. However, they were driven with the same amplifier and equal volume level, for a fair comparison. Therefore, Table 1 gives a qualitative idea of the SPL levels the OPL could generate for pure tones as compared to a dodecahedral loudspeaker, rather than the maximum level it could reach. As said before, this is in fact strongly related to the type of signal excitation, a topic to be further investigated in the future.

Comparing the Q2 levels of the dodecahedral loudspeaker with those of the OPL, we observe that the dodecahedral loudspeaker generates much higher mean SPLs than the OPL. Note also that the SPLs of the dodecahedral loudspeaker tend to decrease with frequency, whereas those of the OPL do the opposite up to 2 kHz and start decaying from there. An explanation for the OPL behaviour could be as follows.

As detailed in Section 2, the SPLs generated by a PAA-based device depend on the square of the difference frequency component (audible sound), $w_{d}^{2}$ (see *K* in Equation (5)). This justifies the level augment with frequency up to 2 kHz. However, at the same time, this increase is somewhat compensated by the resonant behaviour of each ultrasound transducer response. Indeed, the smaller the $w_{d}^{2}$, the higher the ultrasound SPL the transducer emits, since the ultrasonic emitted signal gets closer to the resonance frequency of the transducer. It can then be hypothesized that, despite the nonlinear PAA providing high audible SPLs beyond 2 kHz, these are not powerful enough to compensate for the transducer response, which results in an overall decay of the emitted SPL. This could be fixed by reducing the carrier frequency $f_{c}$ from 41 kHz to that of the ultrasound transducers (∼40 kHz), so that all primary waves (ultrasonic waves) were emitted at a frequency closer to the transducer resonances. The problem in practice is that not all the resonance frequencies of the transducers are exactly the same, and variations of the order of ±1 kHz are common. Adjusting the carrier to ∼40 kHz may therefore result in working below the resonance frequency of some transducers, which would strongly influence the emission at the low frequency range. This is so because the primary waves emitted by each transducer would have different amplitudes, resulting in a worse directivity at low frequencies.

If we next focus on the deviations in Table 1 of Q1, Q3, min and max with respect to Q2, the dodecahedral loudspeaker shows very small variations at low frequencies, whereas those become larger above 1 kHz. This indicates once more that the dodecahedral loudspeaker is losing its omnidirectionallity in the high frequency range. Note, for instance, that, at 8 kHz, the deviations may reach 20 dB at some points (see min-Q2). Indeed, the directivity pattern for 8 kHz in Figure 8 reveals that such strong reductions take place between 30${}^{\circ }$ and 60${}^{\circ }$, and also between 60${}^{\circ }$ and 90${}^{\circ }$. In contrast, the OPL presents smaller deviations in the high frequency range, though, as observed from the DI of Figure 9, it behaves worse at lower frequencies. The OPL clearly improves the performance of the dodecahedral source at high frequencies because all measured SPLs are closer to the mean value Q2.

Finally, we have also computed the acoustic power levels according to the ISO 3745 [42], but again only using the pure tone SPLs of the directivity patterns in Figure 8, without normalization. The acoustic power $L_{w}$ can be computed in free-field conditions as

(8) $$L_{w}=\bar{L_{pf}}+10\log\left(S_{1}/S_{0}\right),$$

where $\bar{L_{pf}}$ is the mean acoustic pressure in a virtual sphere of surface $S_{1}=4\pi r^{2}$ and $S_{0}=1$ $m^{2}$ is the reference surface. To obtain $\bar{L_{pf}}$, one should perform a set of discrete measurements on the sphere surface and then average their acoustic energy. As previously mentioned, in our case, we have taken the measurements performed for the directivity patterns, which correspond to the virtual equator circle. We thus assume, somewhat roughly, that those SPLs are representative enough for the whole virtual sphere, that is, $\bar{L_{pf}}∼L_{\mathrm{mean}}$. The radius *r* of the latter is set to 1.5 m, the distance at which the measurements were performed. The term $10\log(S_{1}/S_{0})$ in Equation (8) therefore adds 14.5 dB to the acoustic pressure levels. On the other hand, note that SPLs were measured emitting a sinusoidal signal of constant frequency. Higher $L_{w}$ values would be produced using, for instance, swept-sine signals thanks to integration in each frequency band. The acoustic power levels for the two sound sources are listed in the last rows of Table 1. In accordance with the Q2 levels in Table 1, the OPL has much lower $L_{w}$ levels than the dodecahedron for all the frequencies.

## 5. Conclusions

In this work, an Omnidirectional Parametric Loudspeaker (OPL) prototype has been designed and constructed. This consists of 750 ultrasound transducers set on the surface of a sphere of radius 0.125 m. The transducers are aligned along the parallels of the sphere, which greatly simplifies their electrical connection and welding. To facilitate the prototype construction, the sphere was split into six pieces, four for the laterals and two for the spherical caps, plus a central piece to guarantee structural robustness, as well as to set the OPL in a microphone stand. The pieces were 3D-printed in plastic material and contained the bases and pin holes for all transducers.

The OPL prototype was tested using pure tones in an anechoic chamber and compared with a commercial dodecahedral loudspeaker. The OPL generated clearly lower SPLs than the dodecahedron for all the frequencies. However, significant differences were found when examining their directivities. For high frequencies, the OPL directivity pattern did not noticeably change with frequency, presenting quite constant SPLs with only some small oscillations. However, in the low frequency range, the OPL did not radiate enough audible SPL, which resulted in larger fluctuations of the directivity pattern. As opposed, the dodecahedral loudspeaker substantially modified its directivity pattern with frequency. Whereas in the low frequency range it was really omnidirectional, for higher frequencies some large dips were produced in the SPLs at certain angles, which moreover strongly depended on the emitted frequencies. The OPL thus exhibited a better performance at higher frequencies than the dodecahedral loudspeaker, although a worse one in the low frequency range. The requirements of the ISO 3382-1 for omnidirectional sound sources were next studied by computing an estimation of the Directivity Index. The dodecahedral loudspeaker fulfilled them, as expected. This was not the case for the OPL, which, despite performing better than the doedecahedron at high frequencies, it failed in the low frequency range. Nonetheless, one should keep in mind that the ISO requirements are defined to suit the performance of dodecahedral loudspeakers, rather than that of an OPL. In other words, it is clearly more permissive for high frequencies than for lower ones. In addition, one should realize that the directivity indices in this work were computed for pure tones. A significant improvement may be expected when using broadband signals instead because of the smoothing effect when integrating over frequency.

In future works, broadband signals such as sine sweeps will be evaluated to get higher sound pressure levels and to characterize the OPL according to current regulations. The behaviour of the OPL when performing acoustic tests in buildings, such as the reverberation time of a room or the airborne sound insulation of a partition, will also be examined.

## 6. Patents

A Spanish patent exists for an initial prototype of the OPL: Patent Number N°201230043, *Omnidirectional sound source based on ultrasonic parametric array technology*.

## Figures and Tables

**Table 1:** Second quartile (Q2), first and third quartile deviations from Q2, minimum and maximum deviations from Q2, energetic mean sound pressure level $L_{\mathrm{mean}}$, and acoustic power $L_{w}$ based on the ISO 3745. All values expressed in dBs and computed from the directivity patterns in Figure 8, for pure tones emitted by the OPL and the dodechaedral source.

Sound Source	Levels	125 Hz	250 Hz	500 Hz	1 kHz	2 kHz	4 kHz	8 kHz
OPL	Q2	43.6	48.2	51.9	56.6	58.6	52.7	45.6
	Q1-Q2	−5.2	−3.4	−1.3	−0.9	−0.8	−1.2	−1.8
	Q3-Q2	4	2.5	2	1.2	0.8	0.9	1.7
	Min-Q2	−29.5	−8	−5.1	−5.5	−3.5	−4.7	−7.8
	Max-Q2	17	14.2	12.5	9.4	4.2	4.4	5.6
	$L_{\mathrm{mean}}$	47.6	50.6	53.4	57.3	58.7	52.9	46.1
	$L_{w}$	62.1	65.1	67.9	71.8	73.3	67.4	60.6
Dodechaedron	Q2	94.9	100.5	94.8	90.1	88.1	83.8	77.1
	Q1-Q2	−0.3	−0.1	−0.1	−0.6	−3.9	−1.7	−1.8
	Q3-Q2	0.3	0.1	0.1	0.5	3.4	2	2.6
	Min-Q2	−0.5	−0.2	−0.2	−1.3	−19	−8.3	−20.4
	Max-Q2	0.5	0.2	0.2	1.5	5.6	3.8	5.9
	$L_{\mathrm{mean}}$	97.2	103.2	98	94.2	93.7	88.3	83.9
	$L_{w}$	111.7	117.7	112.5	108.7	108.2	102.8	98.4

## Abbreviations

The following abbreviations are used in this manuscript:

DI	Directivity Index
OPL	Omnidirectional Parametric Loudspeaker
PAA	Parametric Acoustic Array
PZT	Piezoelectric Transducer
RPL	Regular Polyhedron Loudspeaker
SPL	Sound Pressure Level
USBAM	Upper Side Band Amplitude Modulation
